# ASSOCIATIONS THAT FITNESS AND BODY MASS INDEX HAVE WITH DEFICIT ACCUMULATION FRAILTY

**DOI:** 10.1093/geroni/igad104.2766

**Published:** 2023-12-21

**Authors:** Mark Espeland, Denise Houston, Rena Wing, Felicia Simpson, KayLoni Olson

**Affiliations:** Wake Forest University School of Medicine, Winston-Salem, North Carolina, United States; Wake Forest University School of Medicine, Winston Salem, North Carolina, United States; Miriam Hospital, Providence, Rhode Island, United States; Winston-Salem State University, Winston-Salem, North Carolina, United States; Brown Medical School, Providence, Rhode Island, United States

## Abstract

Lower cardiorespiratory fitness and obesity may accelerate aging processes. The degree to which changes in fitness and body mass index (BMI) may alter the rate of aging may be important for promoting lifestyle changes. We assessed cross-sectional and longitudinal associations that cardiorespiratory fitness and BMI had with a deficit accumulation frailty index (FI). Fitness (based on standardized graded exercise tests) and weight (to calculate BMI) were collected at baseline and year 4 from 3,944 participants, ages 45-76 years, in the Action for Health in Diabetes (Look AHEAD) randomized controlled clinical trial. A validated 38-item FI was used as a marker of aging. Associations between baseline levels and changes in fitness and BMI with changes in FI were assessed using linear models. Both baseline and 4-year changes in fitness and BMI were independently associated with 4-year changes in frailty (all p< 0.001). Participants with the greatest fitness increase and BMI loss tended to have improvements (i.e., decreases) in FI: mean [95% confidence interval] changes -0.019 [-0.024,-0.013]. Those with the greatest fitness loss and increases in BMI tended to have worsening (increased) FI: 0.029 [0.024,0.034]. Associations of 4-year changes in fitness and BMI with FI changes were similar across subgroups based on age, gender, baseline BMI, diabetes duration, and cardiovascular disease history. Increased fitness across 4 years was associated with less FI accumulation independent of baseline fitness. Adults with type 2 diabetes and overweight or obesity may slow aging processes by increasing their cardiorespiratory fitness and losing weight.

